# The anatomy of regression-to-the-mean in simulated epilepsy trials

**DOI:** 10.64898/2026.07.27.26359051

**Published:** 2026-07-31

**Authors:** Daniel M. Goldenholz, Rohan Bhansali, Ted J. Kaptchuk, Brandon Westover

## Abstract

Regression to the mean (RTM) can inflate apparent placebo response in epilepsy trials, but its mechanisms are often conflated. Using CHOCOLATES, we simulated 1,000,000 patients with 36 months of daily seizure counts and simulated placebo trials: 2-month baselines followed by 3-month test periods without treatment effects. Transient worsening (RTM type 1), stricter eligibility thresholds (RTM type 2), reduced sensitivity, and false alarms (RTM type 3) each increased RTM and apparent response. These findings show that placebo-arm improvement can arise from temporary illness, natural variability, measurement error, or mixtures thereof, informing epilepsy trial design and endpoint interpretation.

## Full Text Availability

The license terms selected by the author(s) for this preprint version do not permit archiving in PMC. The full text is available from the preprint server.

